# Supplementary material for: Standard Doses of Cholecalciferol Reduce Glucose and Increase Glutamine in Obesity-Related Hypertension: Results of a Randomized Trial
Source: Int J Mol Sci. 2024 Mar 18;25(6):3416. doi: 10.3390/ijms25063416 (PMC10969945; doi:10.3390/ijms25063416)
Supplement: Supplementary file 1 [file ijms-25-03416-s001.zip › ijms-2885725-supplementary.pdf]

SUPPLEMENTARY MATERIAL

CONSORT flow chart diagram

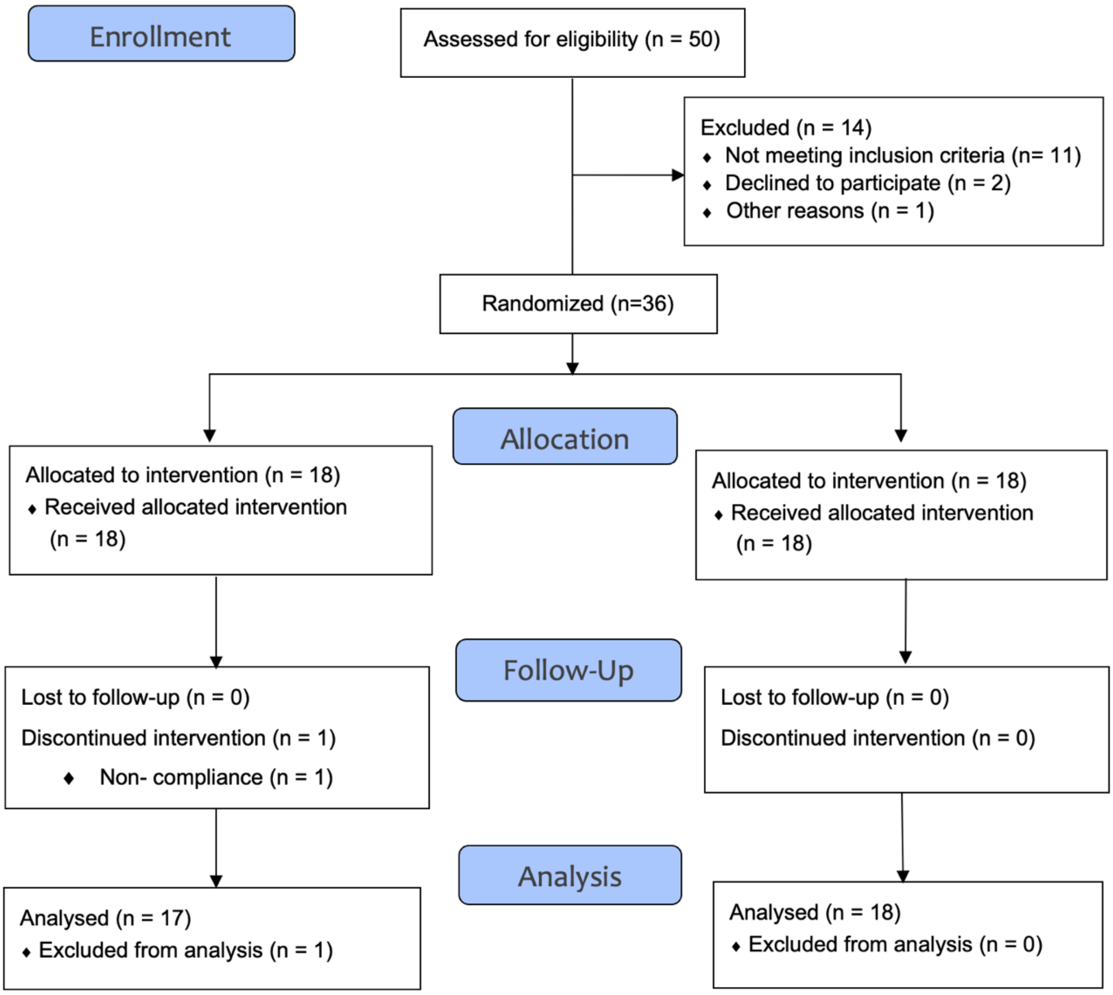

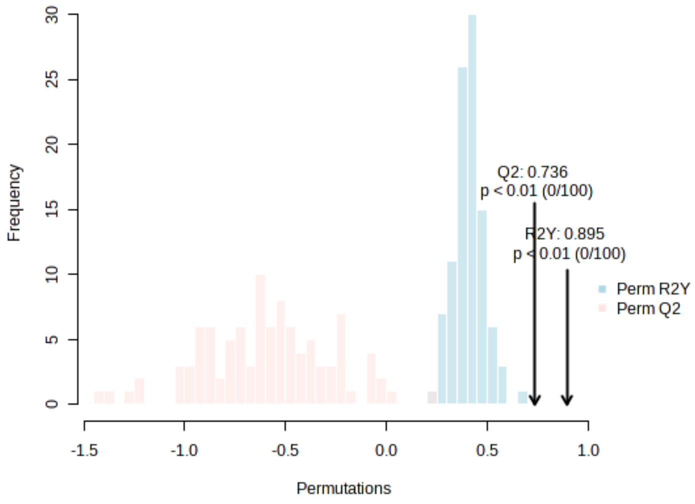

Figure S1. The performance of the Normo0 vs Hyper0 OPLS-DA model was evaluated by the permutation test (n=100). High values of predictive parameter Q^2 and goodness of fit R^2Y (>0.5) as well as $p<0.05$ (difference between original and permutated parameters) indicate robust and reliable predictive model.

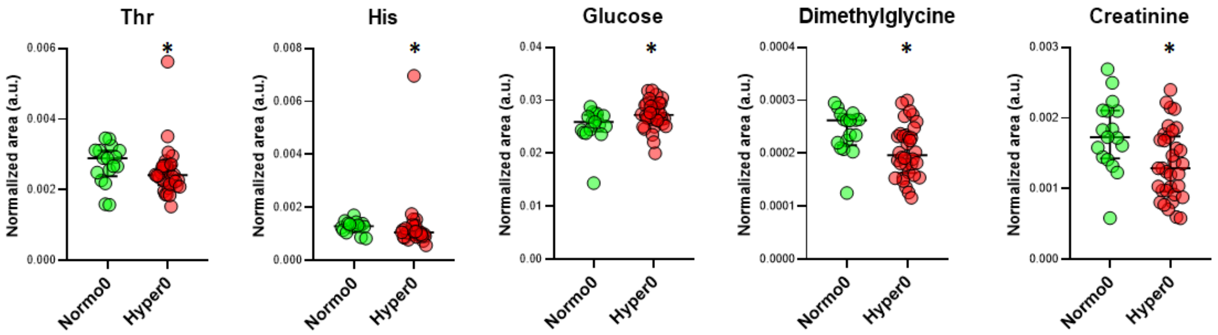

Figure S2: Differentially expressed metabolites between normotensive (Normo0, n=17) and hypertensive patients (Hyper0, n=34) before vitamin D therapy (t0). The differentially expressed metabolites between the 2 groups were selected based only on univariate analysis (p<0.05). Medians with interquartile range are presented. Statistical significance obtained by univariate non-parametric test are denoted as: * p<0.05, ** p<0.001, *** p<0.0001.

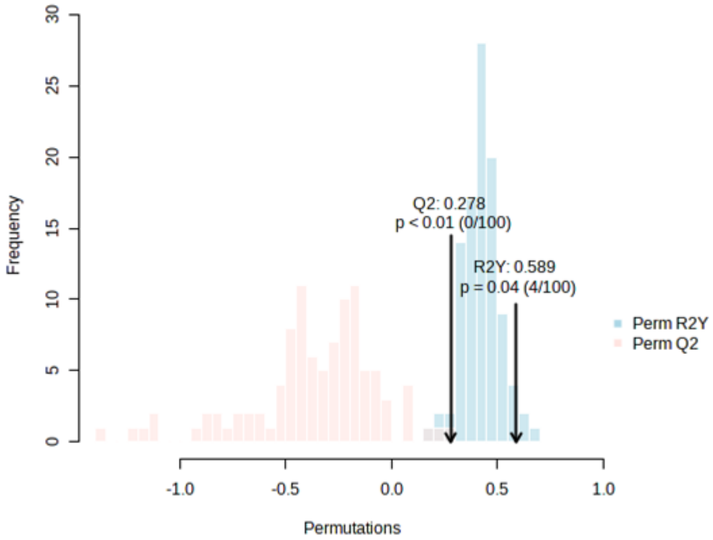

Figure S3. The performance of the HyperAG0 vs HyperAG24 OPLS-DA model was evaluated by the permutation test (n=100). Low values of predictive parameter Q^2 (<0.3) and goodness of fit R^2Y (<0.6) indicate low quality model. Comparison of original and permuted parameters (p<0.05) indicates the model performance is adequate and the model is not overfitted.

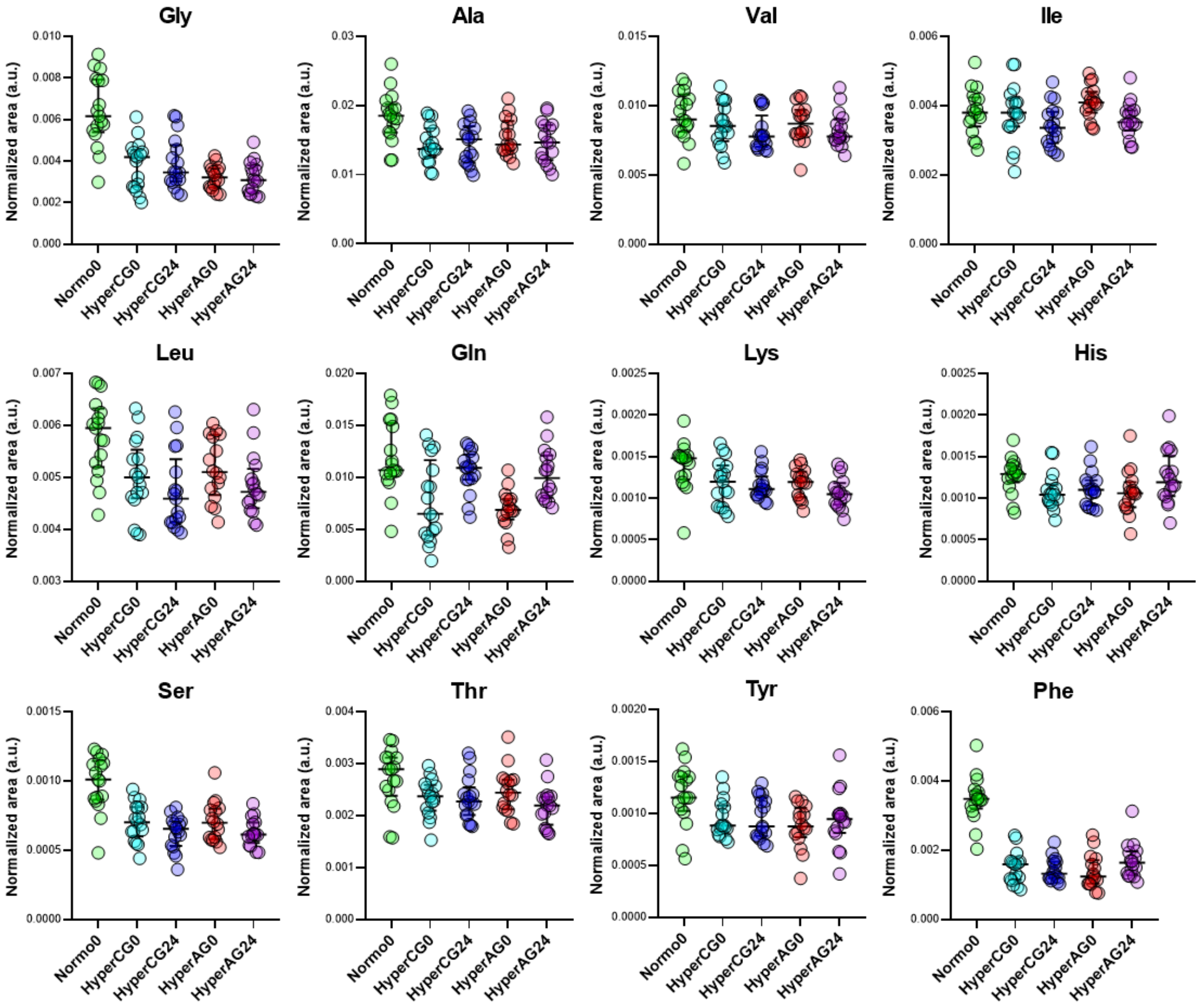

Figure S4: Amino acid levels based on normalized integrals obtained by integration of representative signals in ^{1}H cpmg NMR spectra. Normo0: normotensive control patients (N=17); HyperCG0: control hypertensive patients before the beginning of the study (N=17); HyperCG24: control hypertensive patients after 24 weeks (N=17); HyperAG0: hypertensive active therapy group before vitamin D (N=16); HyperAG24: hypertensive active therapy group after 24 weeks of cholecalciferol (N=16). Medians with interquartile range are presented.

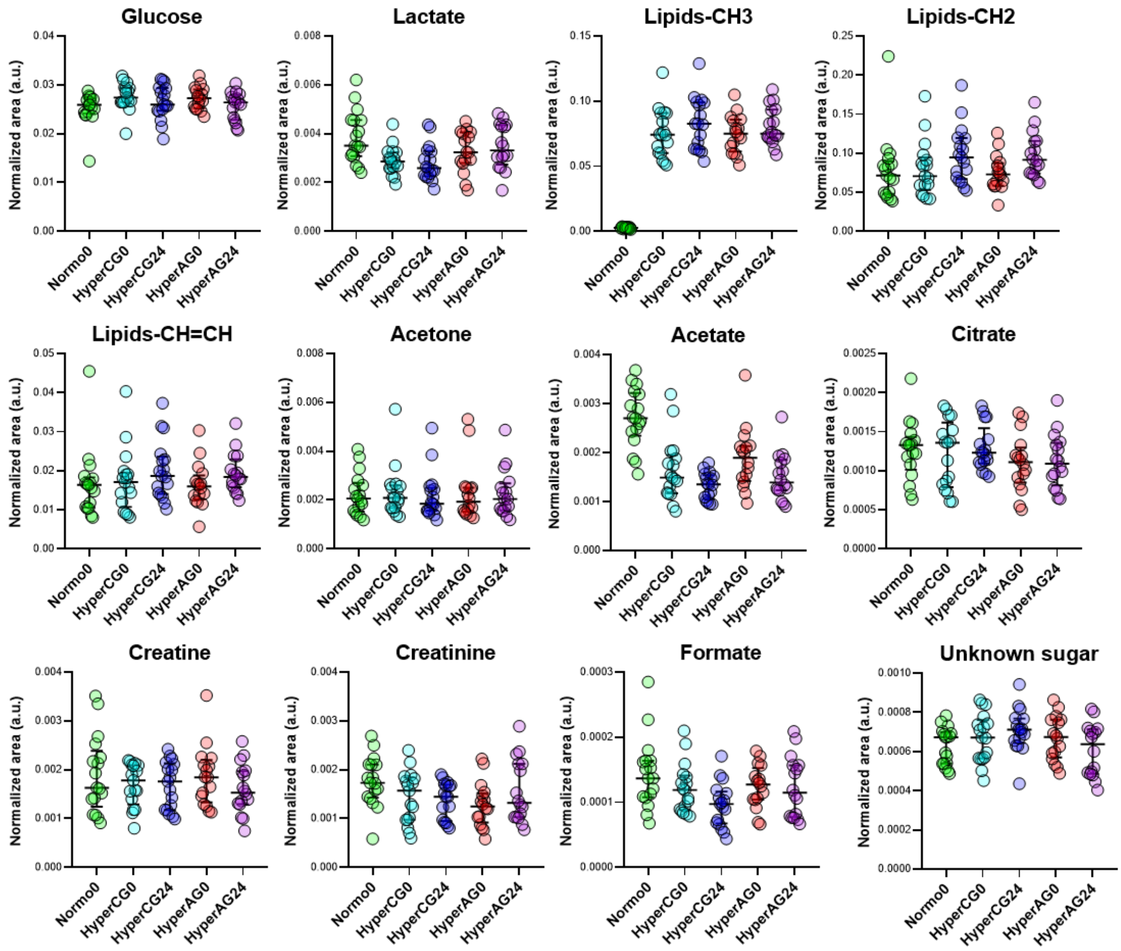

Figure S5: Metabolite levels based on normalized integrals obtained by integration of representative signals in ^{1}H cpmg NMR spectra. Normo0: normotensive control patients (N=17); HyperCG0: control hypertensive patients before the beginning of the study (N=17); HyperCG24: control hypertensive patients after 24 weeks (N=17); HyperAG0: hypertensive active therapy group before vitamin D (N=16); HyperAG24: hypertensive active therapy group after 24 weeks of cholecalciferol (N=16). Medians with interquartile range are presented.

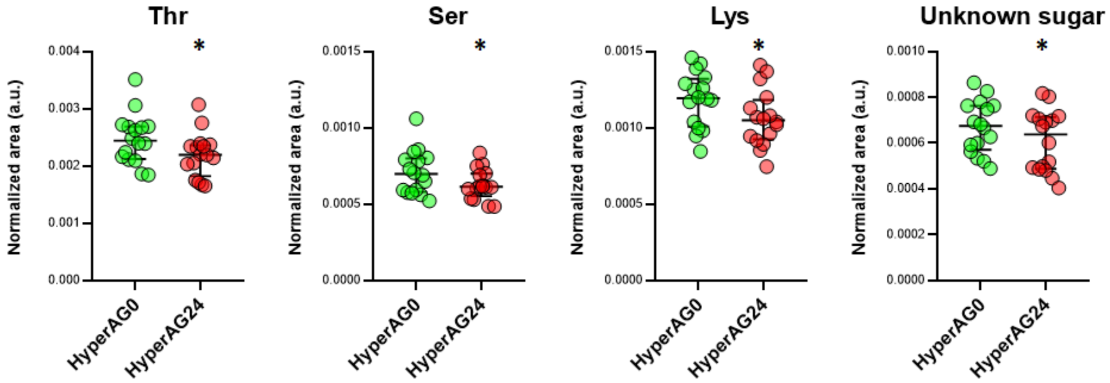

Figure S6: Differentially expressed metabolites between hypertensive group before (HyperAG0, n=16) and after 24 weeks of therapy (HyperAG24, n=16). The differentially expressed metabolites between the 2 groups were selected based only on univariate analysis (p<0.05). Medians with interquartile range are presented. Statistical significance obtained by univariate non-parametric test are denoted as: * p<0.05, ** p<0.001, *** p<0.0001.

Formulas:

Visceral Adiposity Index

Visceral adiposity index	Women	Men
	$= \frac{WC}{36.58+(1.89\ .\ BMI)} \text{ x } \frac{TG}{0.81} \text{ x } \frac{1.52}{HDL}$	$= \frac{WC}{39.68+(1.88\ .\ BMI)} \text{ x } \frac{TG}{1.03} \text{ x } \frac{1.31}{HDL}$

WC: waist circumference

BMI: body mass index

TG: triglycerides

HDL: high density cholesterol
